# Functionalized Cellulose Nanocrystals: A Potential Fire Retardant for Polymer Composites

**DOI:** 10.3390/polym11081361

**Published:** 2019-08-18

**Authors:** Dilpreet S Bajwa, Chad Rehovsky, Jamileh Shojaeiarani, Nicole Stark, Sreekala Bajwa, Mark A Dietenberger

**Affiliations:** 1 Department of Mechanical and Industrial Engineering, Montana State University, Bozeman, MT 59717, USA; 2 Department of Mechanical Engineering, North Dakota State University, Fargo, ND 58108, USA; 3 Mechanical Engineering Department, Western New England University, Springfield, MA 01119, USA; 4 USDA Forest Service, Forest Products Laboratory, Madison, WI 53726, USA; 5 College of Agriculture, Montana State University, Bozeman, MT 59717, USA

**Keywords:** Fire retardant, High-density polyethylene, Nano zinc oxide, Cellulose nanocrystals, Thermal Stability

## Abstract

The flammability of synthetic thermoplastic polymers has been recognized as an increasingly important safety problem. The goal of this study was to evaluate a green and safe fire-retardant system comprising of cellulose nanocrystals (CNC) and zinc oxide nanoparticles (ZnO). CNCs coated with nano ZnO were incorporated in the high-density polyethylene polymer (HDPE) matrix at different concentrations. Fire testing results of different formulations of HDPE containing 0.4 to 1.0% zinc oxide coated CNC exhibited a substantial decrease in the average mass loss, peak heat release rate and total smoke release. The time to ignition exhibited a positive correlation with CNC-ZnO concentration. Modest improvement in the flexural strength and moduli of composites was noticed validating no adverse effects of CNC-ZnO complex. The transmission electron microscopy further confirmed dispersion of nanoparticles as well as the presence of some nanoparticle aggregates in the matrix. The uniform dispersion of CNC-ZnO complex is expected to further improve fire and mechanical properties of polymer.

## 1. Introduction

Synthetic polymers serve as major feedstocks for manufacturing industrial and consumer goods. Flammability of petro-derived polymers has been recognized as an important safety problem. Most widely used thermoplastic polymers such as polyethylene (PE), polypropylene (PP), polystyrene (PS), poly(methyl methacrylate) (PMMA) and acrylonitrile buatadiene styrene (ABS) have poor fire performance in the absence of flame retardants. Their limiting oxygen index is below 19 (average atmospheric oxygen content is 21%). Polyolefins when exposed to high temperatures can degrade, decompose, and yield toxic gases [1,2]. Flame-retardants are generally added to polymeric materials as either additives or reactive materials. Inorganic reactive fillers and halogenated compounds are extensively used to achieve flame resistance in fiber reinforced polymer composite materials [3]. Widely used metal hydroxide require a loading level of 60% or more which leads to a deleterious effect on viscosity and mechanical properties [4]. Inorganic fillers have some drawbacks such as poor compatibility, prone to leaching, and low mechanical properties. Halogenated flame retardants release toxic gases when burned, causing 80% of deaths in fires [5,6]. In addition, a wide array of other toxicant, most notably HBr and brominated phenols/benzenes are produced [7]. These factors suggest a strong need for a safer, effective and environmentally friendly fire retardant system. 

Cellulose, the most abundant organic, renewable material on the earth, has multiple useful properties including excellent mechanical properties, good chemical stability, and high thermal stability [8]. The thermal decomposition pathway of cellulose results in charred structures. Researchers have recently reported complex kinetic models to explain cellulose pyrolysis. It is reported that at low heating rates and low temperature exposure cellulose undergoes dehydration reactions and rearrangement of cellulose results in char formation [9]. Further, a catalytic effect of inorganic salts when combined with cellulose for promoting char is also reported [10]. Recent studies have shown that treating cellulosic materials with nano-sized zinc oxide (ZnO) can provide UV protection [11,12] and enhance flame retardancy [13,14,15]. Specifically, zinc oxide coated cellulose fabrics have shown exceptional flame retardancy as ZnO nanoparticles increase char formation during burning, which acts as a flame suppressant [14,15]. It is reported that metal ions impact the kinetics of thermal decomposition and pyrolysis behavior of cellulose through reducing levoglucosan production and increasing char, water and low molecular compound production [16]. Compared with conventional fire retardants, advantages of zinc oxide include its low price, environmental safety, and high surface reactivity [14]. 

The objective of this research was to evaluate a safe, effective, and environmentally friendly fire retardant for use in HDPE. The central hypothesis of this research was that the incorporation of CNC functionalized by ZnO in polymer matrix would improve their flame resistance by lowering heat release rate (HRR), limiting combustible volatile emissions and/or oxygen supply by charring, shielding or insulating the polymer surface while improving mechanical properties. The mechanism of action is likely to be synergistic between metal oxide-CNC complex and polymer. 

## 2. Experimental

### 2.1. Materials

Analytical grade sodium hydroxide (NaOH) with a molecular weight of 40.00 g/mol was purchased from VWR Chemicals (Randor, PA, USA). Zinc acetate dehydrate, analytical grade with a molecular weight of 219.51 g/mol and a purity of 99.99% was obtained from Sigma-Aldrich (St. Louis, MO, USA). Freeze-dried cellulose nanocrystals synthesized using acid hydrolysis were supplied by USDA-Forest Service (Madison, WI, USA). Analytical grade methanol was purchased from Sigma-Aldrich (St. Louis, MO, USA). Marlex 9006 high density polyethylene manufactured by Chevron Phillips (Woodlands, TX, USA) in the pellet form with a melt flow rate of 6.6 g/10 min and a density of 0.953 g/cm^3^, was used as the base polymer. 

### 2.2. ZnO Nanoparticle Synthesis

ZnO nanoparticles were synthesized by an optimized procedure outlined in previous studies [12,13]. First, 4.39 g of zinc acetate dehydrate was added to 50 mL of methanol, and the solution was heated to 50 °C and stirred for 30 min. Then 1.60 g of sodium hydroxide was added to a separate 50 mL of methanol, and the solution was heated to 50 °C and stirred for 60 min. Next the sodium hydroxide solution was added dropwise to the zinc acetate solution under constant stirring for 30 min. Then stirring was stopped, and the nano-sol was kept at 50 °C for 30 min. Finally, the heat was turned off, and the nano-sol was stirred for another 2 h to create a transparent white gel. 

### 2.3. Coating CNCs with ZnO 

The CNC suspension was created by adding 4% CNC to deionized water and the mixture was homogenized (IKA T50 Homogenizer, Wilmington, NC, USA) for five minutes and then transferred to an ultrasonic probe (Misonix sonicator 3000, Vernon Hills, IL, USA) for five minutes to achieve a stable and clear aqueous suspension. The ZnO-CNC suspension was created by combining 50% ZnO nano-sol and 50% CNCs suspension. The ZnO nano-sol and CNC suspension were mixed together using homogenization until a uniform mixture was obtained. The mixture was then dried in a convection oven at 80 °C for 24 to 48 h until all the solvent had evaporated. The dried mixture was grinded into fine powder (particle size less than 44 micron) using petzel and mortar. For the samples without ZnO, the CNCs were obtained by drying the CNC suspension using the same process.

### 2.4. Nanocomposite Manufacturing

HDPE was blended with either CNC-ZnO or untreated CNCs and directly fed into a twin screw extruder (Leistritz Mic18/Gl-40D, Nuremberg, Germany) to evenly incorporate the ZnO coated CNCs into the HDPE. The polymer processing temperature of extruder varied from 160, 193, 198, 204, 205, 207, 216 °C (eight zones, feed throat to die end) respectively, with a speed of 150 RPM. Compression molding was used to manufacture 4 × 100 × 200 mm^3^ (thickness, width, length) sheets using a heated hydraulic press (Carver 3856, Wabash, IN, USA) at a temperature of 177 °C and a pressure of 2 metric tons for 25 min. Table 1 shows the materials.

### 2.5.Characterization and Measurement

#### 2.5.1. Transmission Electron Microscopy Analysis

The particle shapes, sizes and distribution of ZnO nano-sol, CNC suspension, and CNC-ZnO suspension were analyzed using a transmission electron microscopy (JEOL Inc., Peabody, MA, USA) operating at 2 kV. The CNC and nano ZnO suspensions were placed using an aqueous dispersion on a 300 mesh carbon-coated copper grids. The samples were subsequently stained by phosphotungstic acid to enhance the microscopic resolution.

#### 2.5.2. Fire Testing 

The flammability and flame resistance tests were conducted by USDA Forest Service, Forest Products Laboratory in Madison, WI and University of Dayton Research Institute, Dayton, OH. Mass loss calorimetry was conducted in accordance with ASTM E2102 at the Dayton Research Institute and Cone calorimetry conducted according to ASTM E1354 at the Forest Products Laboratory (FPL). Cone Calorimeter experiments were conducted on a FTT Dual Cone Calorimeter at one heat flux (35 kW/m^2^) with an exhaust flow of 24 L/s using the standardized cone calorimeter procedure. Samples were oriented horizontally with no grid. 

#### 2.5.3. Mechanical Properties Testing

Flexural testing was conducted in accordance with ASTM D790. Instron, a universal testing machine equipped with a 2 kN load cell was used. Samples sheets were cut into 4 mm thick by 12 mm wide strips to be used as test specimens. 

## 3. Results and Discussion

### 3.1. Transmission Electron and Scanning Electron Microscopy 

**Figure 1:**
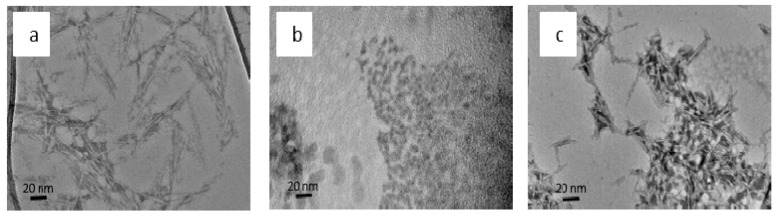
TEM Images of (**a**) CNCs, (**b**) ZnO Nanoparticles, and (**c**) ZnO Coated CNCs.

**Figure 2:**
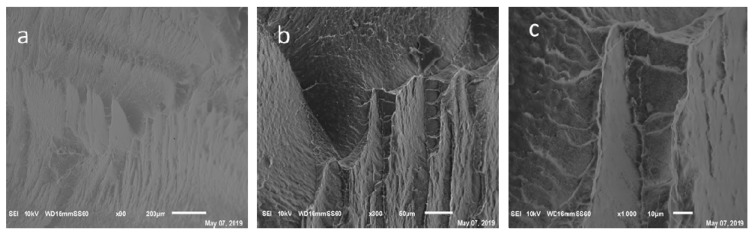
SEM Images of HDPE, CNC-ZnO composite at different magnifications: (**a)** 90×, (**b)** 300×, and (**c)** 1000×.

Transmission Electron microscopy (TEM) verified that the ZnO particles and CNCs were on the nanometer scale in aqueous suspensions. The CNCs were long, slender rod shapes, and the ZnO nanoparticles were spherical (Figure 1). When the ZnO particles and CNCs were combined, the ZnO particles encapsulated or surrounded the CNCs as desired, but some agglomerates were also noticed. The scanning electron microscopy (SEM) images of the HDPE composites with 1% CNC-ZnO at three different magnifications show a reasonable dispersion of nanoparticles in the composites (Figure 2). 

### 3.2. Flame Testing

The fire testing results showed that addition of 1% CNC-ZnO lowered the average mass loss, peak heat release and total smoke release by 9%, 11.5% and 14.7% respectively compared to 100% HDPE (Table 2). The peak heat release was reduced by 18% when 0.4% CNC-ZnO were added to HDPE. Similarly time to ignition increased by 25% with 1% addition of CNC-ZnO complex. The decrease in average mass loss and peak heat release rate can be attributed to the incipient development of char caused by the flame retardant. 

**Figure 3:**
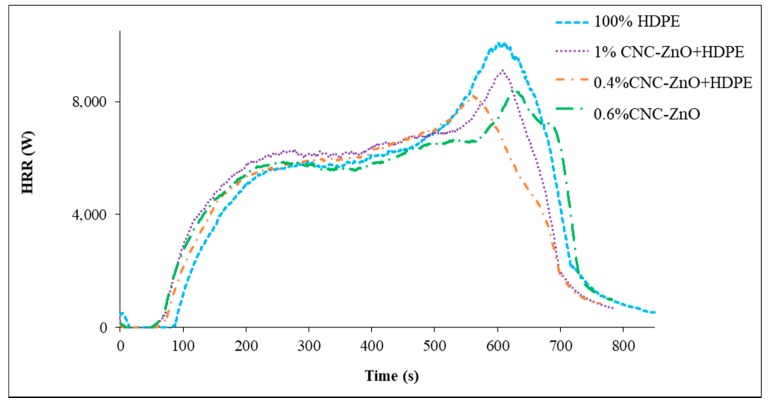
Heat Release Rate of HDPE and CNC-ZnO Formulations.

**Figure 4:**
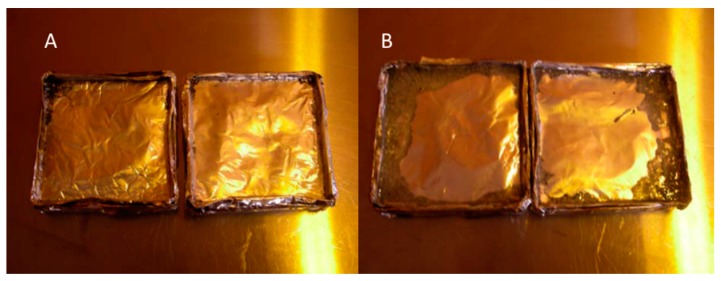
Final char of composites, A. Virgin HDPE and B. HDPE with 0.6% CNC-ZnO.

The improvement in the flame resistance of polymer can be attributed to ZnO coated CNCs char on the outer surface of the polymer composite, which shields the polymer surface by reducing initially combustible gases as fuel for the flame ignition and growth, and later, the low char surface combustion rates with oxygen penetration in the porous char. This causes the sample to burn more slowly. Increased piloted time to ignition is the result of reduced pyrolysis rates from the polymer composite with the charred surface layer occurring prior to ignition in order to reach a critical mass loss rate (with a sufficient volatile heat of combustion) for ignition.” All these observations are in agreement with recent reports suggesting fire retardant behavior of nano ZnO and metal ions [15,17]. Figure 3 shows the heat release rate of each sample over the duration of the testing. All of the samples follow a similar trend until about 600 s into the experiment when it can be seen that the ZnO coated CNCs restrict the burning to a lower peak heat release rate. The final chars of virgin HDPE samples and 0.6% CNC-ZnO are shown in Figure 4. The final chars (Figure 4, right) show no residue in virgin HDPE other than a small amount of black ash in the corners of the aluminum foil sample holder. In case of HDPE sample with 0.6% CNC-ZnO, there is some residue remaining with black/grey ash at the corners. The higher amount of char for HDPE composites with CNC-ZnO further supports the fire retardant behavior of the CNC-ZnO complex. 

### 3.3. Mechanical Properties Testing

**Figure 5:**
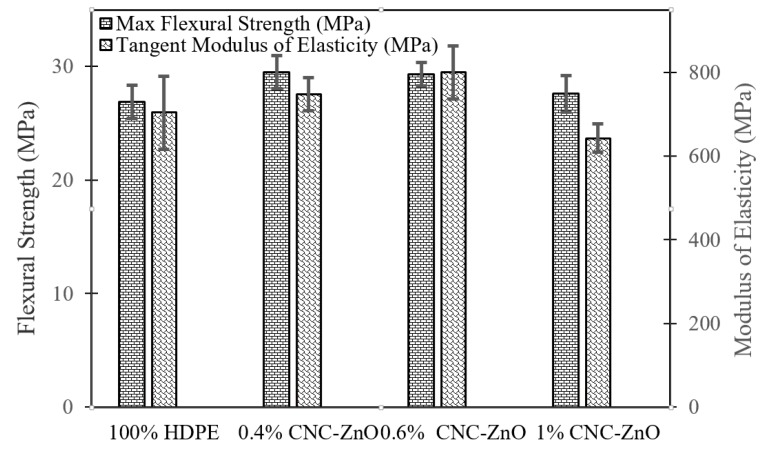
Mechanical Properties of HDPE and CNC-ZnO Formulations.

The addition of ZnO coated CNCs led to some changes in the flexural strength and modulus of elasticity compared to pure HDPE. Figure 5 shows the mechanical property changes of each formulation. An improvement in moduli and strength was noticed, with the latter showing more gain. Typically, the addition of CNCs to a polymer matrix would lead to an increase in flexural strength and modulus of elasticity. Formulation with 0.6% CNC-ZnO exhibited the highest gain in the mechanical properties compared to 1% CNC-ZnO formulation. This can be attributed to better dispersion of the nano particles in the matrix. The lower properties of 1% CNC-ZnO formulation are likely due to poor dispersion of the CNCs in the polymer matrix. Agglomerates of CNCs would create stress concentrations that would lead to earlier fracture. Uniform dispersion of CNC-ZnO in polymer matrix is expected to further improve flame retardancy and mechanical properties. In general, addition of the ZnO coated CNCs did not have any adverse effects on the strength of the polymer. 

## 4. Conclusions

The study found that addition of ZnO coated CNCs to HDPE decreased the average mass loss, peak heat release rate and total smoke release compared to pure polymer. An increase in the ignition time was observed. The results verified that CNC-ZnO nanoparticles can act as a flame retardant when introduced into HDPE polymer matrix. The ZnO coated CNCs create a layer of char around the outer surface of the polymer, which helped to lower the mass loss rates of the polymer and result in slower burning, particularly near the fuel burnout peak that may be affected the most by the stable char. The presence of CNC-ZnO in HDPE marginally improved the moduli and strength properties. The variability in the flame resistance and mechanical properties is due to uneven dispersion of nanoparticles and some charring of CNC during processing of the composites. Overall, the results showed the potential of CNC-ZnO nanoparticles as a safe, fire retardant system in HDPE composites. 

## Figures and Tables

**Table 1:** Labels and Compositions Details of Examined Samples.

Sample code	HDPE (wt %)	CNC (wt %)	ZnO (wt %)
HDPE	100	0.0	0.0
0.4% CNC-ZnO	99.2	0.4	0.4
0.6% CNC-ZnO	98.8	0.6	0.6
1% CNC-ZnO	98.0	1.0	1.0

**Table 2:** Burn Characteristics of HDPE and CNC-ZnO formulations.

Formulation	Time to Ignition (s)	Average Mass Loss (g/m^2^s)	Peak Heat Release Rate (kW/m^2^)	Total Smoke Release m^2^/m^2^
HDPE	64	22.9	1140.9	1512
0.4% CNC-ZnO	70	21.6	930.6	-
0.6% CNC-ZnO	64	20.8	951.6	1415
1% CNC-ZnO	85	18.3	1030.5	1381
